# African Primary Care Research: Quality improvement cycles

**DOI:** 10.4102/phcfm.v6i1.598

**Published:** 2014-04-24

**Authors:** Claire van Deventer, Bob Mash

**Affiliations:** 1 Department of Family Medicine and Rural Health, University of the Witwatersrand, South Africa; 2 Division of Family Medicine and Primary Care, Stellenbosch University, South Africa

## Abstract

Improving the quality of clinical care and translating evidence into clinical practice is commonly a focus of primary care research. This article is part of a series on primary care research and outlines an approach to performing a quality improvement cycle as part of a research assignment at a Masters level. The article aims to help researchers design their quality improvement cycle and write their research project proposal.

## Abstrait

**La recherche sur les soins de santé primaire en Afrique: les cycles d'amélioration de la qualité**. L'amélioration de la qualité de la prise en charge médicale et l'application des données probantes à la pratique clinique font couramment l'objet de l'attention de la recherche portant sur les soins de santé primaire. Cet article fait partie d'une série dédiée à la recherche sur les soins de santé primaire et définit une approche à la réalisation d'un cycle d'amélioration de la qualité, dans le cadre d'un travail de recherche entrepris au niveau du Master. L'objectif de cet article est d'aider les chercheurs à élaborer leur cycle d'amélioration de la qualité et à rédiger leur proposition de projet de recherche.

## Introduction

This article outlines the study design for quality improvement (QI) cycles when performed as a research project. QI cycles can be seen as a form of translational research:

Translational research transforms currently available knowledge into useful measures for everyday clinical and public health practice. Translational research aims to assess implementation of standards of care, understand the barriers to their implementation, and intervene throughout all levels of health care delivery and public health to improve quality of care and health outcomes, including quality of life.^1^

Translational research is important in the context of primary care because there is often more benefit for the population served from ensuring the effective implementation of what is already known than from developing new therapies or technology from basic science. Performing a QI cycle in the discipline of family medicine and primary care is therefore recognised as a legitimate research activity that provides new knowledge on how to improve the quality of care in that context.^2^

One of the roles of family physicians is often that of clinical governance, which at its heart is also an approach to improving the quality of clinical care. Many health services have embedded QI strategies as a routine part of the organisational culture and policy.^3, 4^ There are publications from South Africa, Scandinavia, the Netherlands, the United States of America and many others, where the long-term advantages of a process of QI in healthcare have been recognised.^4, 5, 6, 7, 8, 9, 10, 11^ Skills in QI are therefore a necessary competency for family physicians. When performed as part of a research project, however, there must be greater attention to scientific rigour, especially with regard to ensuring sufficient sample size and applying appropriate statistical methods of data collection and analysis or using the best qualitative methods, should this be the preferred route.

There are many reasons to start a QI project:^12^

- Bridging the gap between evidence, policy and practice.
- Improving the patient's experience of care.
- Educating and training health workers in terms of best evidence-based practice.
- Improving teamwork and motivation to improve quality of care.
- Improving management and accountability.
- Improving financial planning and budgeting of healthcare services.
- Identifying further research questions.

## Historical background

The earliest examples of quality in health may be seen in Florence Nightingale's interventions in the Crimean war^13^ in 1854, the British farming industry in the early 1900s^14^ and Donabedian's contribution in the 1960s toward the framework of quality in health that comprises structural, process and outcome elements of quality.^15^

Quality improvement was studied as an industrial process in 1931 by Shewhart.^16^ His concepts included identifying customers’ needs, reducing variation in processes and minimising unnecessary supervision. Influenced by Shewhart's work, Deming recognised QI as a primary driver for industrial success and subsequently introduced these methods to post-World War II Japanese engineers and executives.^17^ Applied strategically, these methods produced considerable growth in the Japanese automobile industry and became recognised worldwide as QI methods.^18^

Based on the above influences, Taiichi Ohno, a Toyota Motor Corporation engineer, revolutionised thinking about process inefficiency or ‘waste’ in the early 1950s, leading to the creation of the Toyota Production System (TPS).^19^ Application of the TPS resulted in the use of the term *lean* in many industries, including healthcare.^20^ Lean methodology is driven by the identified needs of the customer and aims to improve processes by removing non-value-added activities.

Non-value-added activities, also referred to as waste, do not add to effectiveness, financial success or the customer's experience; and the customer is often not willing to pay for them. Seven different types of waste have been identified, as shown in Table 1.

**TABLE 1:** Types of organisational waste and examples from healthcare.

Types of waste	Examples for healthcare
Overproduction	Pre-mixing drugs or performing laboratory tests ‘just in case’ they might be useful.
Wasted inventory	Using beds for patients who are just waiting for test results, getting patients back weekly to see a clinic nurse for tuberculosis (TB) treatment.
Rejects/defects	Mislabelling laboratory specimens, using broken or faulty equipment.
Wasted motion	Having only one emergency trolley between many wards, resulting in having to spend time hunting for equipment to perform a common procedure.
Waiting/delay	Long queues or waiting times, waiting unnecessarily long for medication, results or to see a healthcare worker.
Waste with processing	Time spent on completing irrelevant paperwork, spending time on intravenous treatment when oral is equally effective.
Waste with transport	Transporting patients to a referral hospital for treatment or investigations that should be performed locally, transporting specimens to a laboratory when point-of-care testing would be equally effective.

## Understanding the concept of ‘quality’ in healthcare

The WONCA Working Party on Quality in Family Medicine defines quality as: ‘The best health outcomes that are possible, given available resources, and that are consistent with patient values and preferences’.^21^ Quality of care can be defined from different perspectives with different priorities – the patient, the primary care provider, the fund manager or the policy maker. Quality of care can also be evaluated at different levels of the health system, from the individual person, to a whole health centre or clinic, to a subdistrict or district, or even at a national level.

The focus of quality improvement in primary care is often on the quality of clinical care within the consultation for specific conditions such as HIV, diabetes or hypertension. However, there are additional dimensions that should be considered. The performance of the health service as a whole may also be relevant:

- Accessibility:^22^ Are patients capable of getting healthcare services when needed, in terms of both geographic and financial barriers? Is care organised in a way that maximises physical access (eg. convenient times, ramps for patients with disabilities) and convenience for patients? Is the clinic able to cope with the volume of patients? Is care equally accessible to all? Is there a financial barrier for certain levels of health (eg. MRI scans for state patients)? Negative attitudes of staff members may also prevent optimal access.
- Acceptability:^23^ How satisfied are patients with their care? Are there cultural or spiritual issues that are ignored?
- Continuity:^24^ Is care organised in such a way that there is retention of information between visits and over time as well as some longitudinal continuity with at least a team of the same primary care providers?
- Coordination:^22^ Is there cooperation between the community and clinic, within the multiprofessional team at the clinic or health centre and between the clinic and the referral hospital?
- Comprehensiveness:^24^ Does the facility offer the full range of services needed by this community?
- Effectiveness:^22^ ‘Doing the right thing’ is in many ways addressed directly through the QI cycle in the structural, process and outcome criteria that are set and based on the best available evidence.
- Efficiency:^23^ ‘[*T*]he balance between the level of resources in the system used to treat patients to come to certain outcomes’. From the patient's point of view, allocative or productive efficiency relates to maximising their health outcome whilst minimising the costs and time spent for the patient. Technical efficiency implies that the system cannot reduce its use of resources any further without also eroding its ability to treat patients or deliver on the required outcomes. Performance efficiency may also relate to issues such as the number and duration of consultations, rationale for use of medication or number of new referrals.
- Equity:^23, 25^ This is ‘[*t*]he absence of systematic and potentially remediable differences in health status across population groups’ and is entrenched in the South African National Core standards as part of the involvement and fairness toward patients when related to issues of quality.

There are a number of confusing concepts regarding QI. Whilst quality assessment (audit) is the process of evaluating the current level of performance, QI is a process of change and improvement that uses the audit as a source of information. Quality assurance requires both these aspects in order to assure that good standards of care are maintained through repeated cycles of assessment and improvement.^26^

When looking at quality as part of a research project for a Master's degree, it is usually necesssary to go beyond just auditing the current practice (what is happening). At the same time, it is usually impractical to perform continuous quality assurance over several years. Quality improvement is therefore the most usual goal of such a project, implying that a full QI cycle should be completed in which the current quality of care is audited, change to improve the quality of care is implemented and the effect of this intervention is reaudited in order to determine if there has been an improvement.

## Choosing a topic

In your research proposal, the argument for the social and scientific value of your chosen topic will be made in the introduction to the proposal. The topic should be of importance to patients’ health or wellbeing, be amenable to change, be relatively common with sufficient patient numbers and be practical to study in terms of available time and resources. Topics may be chosen due to the awareness of a gap between evidence and practice, experience of a critical adverse event such as a maternal death, unacceptable variation in practice between people or facilities, or because data is being collected routinely and can be used to identify health priorities as well as to monitor progress. There may be problems that are important for patients, healthcare providers or other partners in healthcare such as policy makers or carers.

## Aim and objectives

In QI, the research question is usually a ‘How?’ question. An example is ‘How to improve the quality of care for congestive cardiac failure at a given Community Health Centre?’ and the aim, therefore, could be expressed as ‘To assess and improve the quality of care for congestive heart failure at a given Community Health Centre’. When writing the objectives one should avoid describing the methodological steps of the QI cycle and rather describe the objectives that you want to achieve as a result of these steps. For example:

- To assess the current quality of care for congestive cardiac failure.
- To plan and implement changes to improve the quality of care.
- To determine if these changes are associated with a measurable improvement in the quality of care.

## Methods

### Study design

**FIGURE 1:**
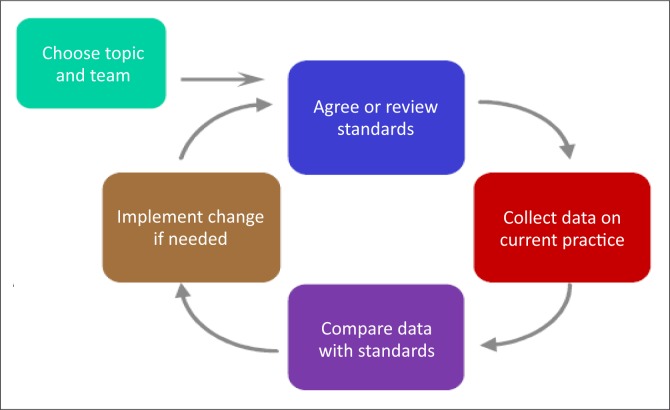
The quality improvement cycle.^27^

The study design for QI is usually a series of steps that can be conceptualised as a cycle. When writing the research proposal, it may be clearer to describe each of these steps under a number of subheadings (see below), rather than trying to force the description of the design into the traditional subsections of the methods. The overall design of the cycle can be described in the study design section of the proposal and a diagram to illustrate it is often helpful (Figure 1).

### Setting

In your research proposal you should describe the clinical setting for the QI cycle with some detail regarding the current service for the topic in question. You should also describe the population served by the facility, in order to provide a context for understanding the topic and for interpreting the data.

### Selecting a team

Quality improvement is usually a team effort, as ownership of the problem and process by the people involved is more likely to lead to change in practice than when it is perceived as criticism by an outside researcher or individual. In a study done with a QI team regarding management of diabetic patients in a primary care setting, the following was found:

Success was more likely in teams in which: the GP or nurse felt personally involved in the audit; they perceived their teamwork as good; they had recognized the need for systematic plans to address obstacles to quality improvement; and their teams had a positive attitude to continued monitoring of care. A positive attitude to audit and a personal interest in the disease were not associated with improvement in care.27

Set up a meeting to explain the concept of what QI is, what it is not (policing, criticism), that participation is crucial and explain the process. Involve all the people that could improve the situation and assist in maintaining the long-term outcome. The team will usually consist of key leadership within the facility. Consideration should be given to the involvement of patient advocates. The team should also ensure that they give and receive feedback from the broader healthcare team during the process.

In your research proposal, describe how you will establish your team, what categories of people you intend to be a part thereof and why you have purposefully selected these categories. The team members each need to add value to the project and will influence both the outcome and the sustainability thereof.

### Agree on or review standards of care

Target standards for QI are usually a combination of a measurable and clearly-defined criteria and a level of desired performance.^22^

Criteria are often aspects of care and are chosen with respect to the structure, process or outcome of care (see Table 2 for examples):

- Structure would include the available physical amenities, human resources, equipment, medication and educational materials.
- Process includes all the healthcare activities that take place during the patient's progress through the facility, including the consultation.
- Outcomes are the end results of the care received, for example the successful treatment of a condition, level of control achieved, or avoidance of complications.

**TABLE 2:** Examples of criteria for cervical screening.

Structure	Criteria
All consulting rooms used by professional nurses have specula available in small, medium and large sizes.	Structural criteria
Patients seen have all their details entered into the cervical screening register.	Process criteria
Professional nurses have performed 10 cervical smears per week during the previous month.	Process criteria
Women over 30 years of age have had at least one documented cervical smear in the previous three years.	Outcome criteria

Criteria should be evidence based and derived from a synthesis of the evidence, ideally in the form of a relevant and valid guideline.^22^ In the absence of such a guideline, systematic reviews or other types of studies can guide the selection of criteria. The evidence should clearly make the case for why this criterion is linked to the quality of care for this specific condition or topic. Criteria should also be selected that are measurable given available time and resources and should be amenable to change. The number of criteria should also be realistic – not everything needs to be measured. The criteria that best depict the quality of care and which are most sensitive to improvement should be chosen. Criteria should, however, include all the key areas and focus particularly on where the team anticipates the problems with quality to be.

A standard can be agreed upon. This can be based on the results of previous QI cycles or on national or international guidelines or simply agreed to by the project team as a reasonable goal for QI. They should be realistic (for example, levels of 100% are seldom achieved) and yet promote the improvement of quality over time. Table 2 gives examples of criteria for cervical screening. The standard here could possibly be ‘60% of women qualifying for a papsmear, receive one’, based on the national target of 54%.^28^

In your research proposal, you should describe the sources of evidence that you will use with your team and the process that you will follow to set these standards. If this process has not yet happened it may be helpful to at least give some examples of likely standards that may be used.

### Collect and analyse data on current practice

When performing QI as part of a research project, it is important to ensure a sufficient sample size if you are relying mostly on quantitiave methods. Remember that you intend not only to achieve a representative sample of your target population (e.g. all women over the age of 30 years that require cervical screening or all patients with type 2 diabetes), but also to compare the results at baseline with the results at follow up to determine if there is a statistically-significant improvement in care (this will require sufficient power in your sample size). Sometimes you can demonstrate a statistically-significant improvement even when your standard is not achieved. A suitable sampling strategy must also be used to ensure that you obtain an unbiased sample of your target population. See the article on surveys and questionnaires for help with sample size calculation and sampling strategies.

Common sources of data to measure your criteria in primary care include patients’ records, registers (e.g. TB and mental health registers) or routinely-collected statistics on healthcare from the clinic. Data can be collected prospectively, as patients are seen or, more often, retrospectively, from data that has already been collected.^24, 25^ A data collection tool may be developed and data captured in an Excel spreadsheet for further analysis. See the article on quantitative data analysis elsewhere in this series.

Qualitative data is sometimes also collected to complement the measurement of criteria and to enable a deeper understanding of how to improve quality. For example, in-depth interviews or focus group interviews with patients can provide valuable insights from their perspective. Patient satisfaction and experience can also be measured by means of a questionnaire. This baseline data can then be analysed using descriptive statistics or qualitative data analysis as described in other articles in this series. Make sure that you have analysed your data so as to measure the exact criteria that you defined previously.

In your research proposal, therefore, you need to describe your sample size calculation, sampling strategy, data collection process and analysis of the data.

### Compare data with standards

The results of this baseline audit can then be compared to the standards set previously by the team. In essence, this makes the discrepancy between actual and desired practice visible to the team. The researcher should aim to facilitate reflection on these results with the whole team with the goal of reaching a consensus on what has been learnt regarding the strengths and weaknesses of current quality.

In your research proposal you should describe how you will facilitate this process and any specific techniques that you will use. A few useful techniques are described below:

#### Process mapping

**FIGURE 2:**
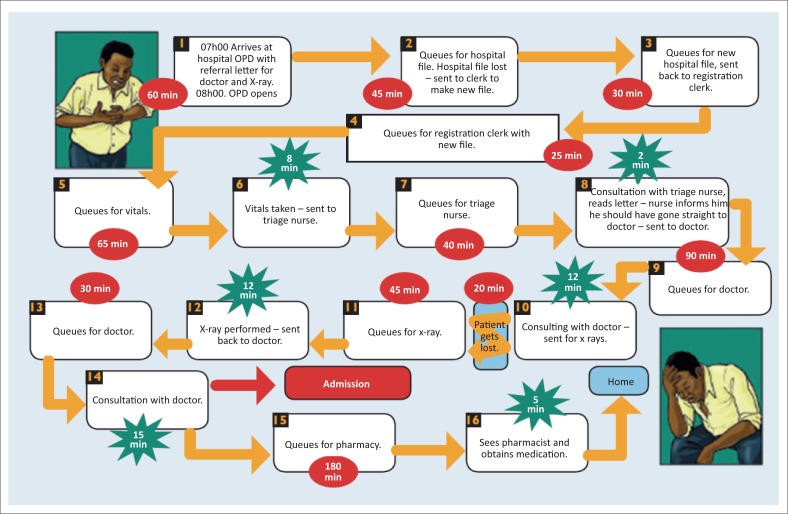
Example of process mapping diagram.^27^

Process mapping is an objective assessment of systems or processes in order to identify road blocks and bottle necks within the processes such as long waiting times in a clinic, or the process from antenatal booking to delivery of a baby.^29^ The team can do this as a theoretical exercise where the current situation is analysed. On individual post-it notes, a process is mapped out as shown in Figure 2. Each step is documented starting on the left, with arrows between the post-it notes. At each note, the people involved should be documented. The time taken should be annotated in red if the data point is non-value-added or in green if it is value-added. This leads to objective and constructive discussions relating to bottlenecks, unseen problems, obvious solutions and ongoing monitoring systems. Patient shadowing would require team members to physically accompany a patient through all the processes and note everything that occurs and the time taken.

#### Fishbone and/or root cause analyses

**FIGURE 3:**
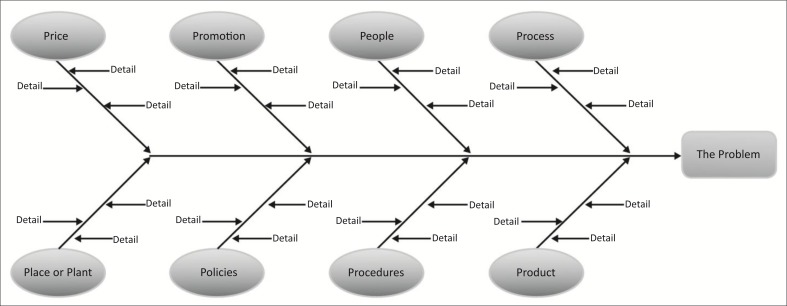
Fishbone diagram for root cause analysis.

Process mapping is often used together with other tools such as the fishbone or root cause analyses shown in Figure 3.^29^ The fishbone assists in separating and organising the contributing factors towards the root cause of the problem.

**FIGURE 4:**
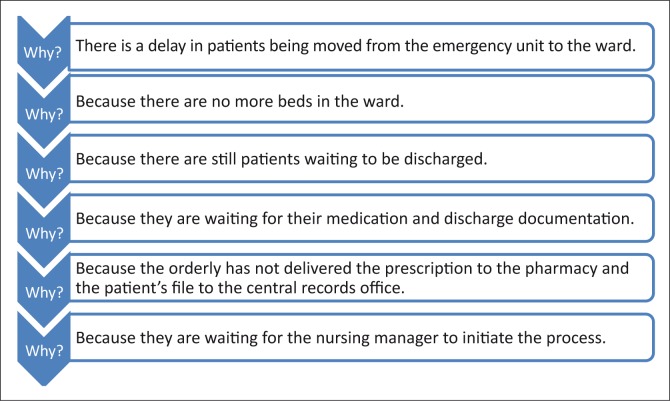
The 5 Whys used in root cause analysis.

The ‘5 whys’ is another way of doing a root cause analysis, by asking the question ‘why?’ until one reaches a common denominator, as is shown in Figure 4.^29^

### Plan and implement change

The reflective and analytical process described above should lead naturally to reflection within the team regarding how to change current practice and improve quality. One should ensure a thorough reflection on the real issues and not jump to conclusions too quickly on what to change. In your research proposal, you should also describe how you will facilitate this process.

As with all planning processes, it should be made very clear what has been agreed to, who is responsible and what the timeframe is for implementation. The plan and the implementation thereof need to be SMART (Specific, Measurable, Achievable, Realistic and Time-framed). Small-scale or individual Plan-Do-Study-Act (PDSA) cycles can be used to pilot suggested changes prior to full-scale implementation within the facility.^30, 31^ If there is a need for further training in order to implement the plans, this should also be identified and organised. If there is a need for additional resources or input from higher up the organisational ladder, then this should also be identified and a strategy agreed on for engaging with the relevant managers. The results of the audit, interpretation and plans for change should be communicated to the rest of the staff in a way that encourages further feedback, reflection and ownership.

The team should plan the implementation carefully as well as how they will monitor and follow up on progress. Regular meetings are helpful with regard to tracking the progress of the implementation.

Your research proposal should describe how you will facilitate this process; how the plans will be implemented, monitored and reinforced over time; and how long you anticipate it will take before they result in a measurable improvement in quality.

### Repeat the cycle

After a defined period of time the audit should be repeated to re-measure the criteria. This period is usually six months to one year in order to allow time for the intervention to be effective and this should be anticipated in the timeline for your research proposal. Ideally, the same patients or their medical records should be included in the re-audit so that you are measuring change in the same group of people over time. This makes comparative statistical analysis possible. In your research proposal, you should describe when and how you will re-audit the criteria.

In your final research assignment, it may be useful to report on any further reflections of the team on the final results, what has been achieved and what must be focused on next. You should report on what worked and what did not work in terms of changing clinical practice and how this might be useful to other practitioners. It may also be helpful to reflect on whether the criteria you used need to be modified or adapted in some way for future use. The propositional knowledge generated by the QI cycle includes a report on the quality of care in the study context and on how care can be improved in this context.

Many QI cycles performed for research purposes are once-off projects. Sustaining the change should be considered from the beginning with ongoing QI cycles and monitoring systems built in after the research project has finished.

## Conclusion

This article describes the QI programme design and elaborates on the key steps involved in a QI cycle, performed as a translational research study and, in particular, advises on how to write a research proposal for such a study. If a researcher chooses a relevant and important topic, gathers an appropriate team and has evidence-based target standards and innovative assessment methods, these should result in a SMART plan, which can be implemented and reassessed and which can lead to actual change in clinical practice.
